# Seizure Onset Zone Detection Based on Convolutional Neural Networks and EEG Signals

**DOI:** 10.3390/brainsci14111090

**Published:** 2024-10-29

**Authors:** Zhejun Kuang, Liming Guo, Jingrui Wang, Jian Zhao, Liu Wang, Kangwei Geng

**Affiliations:** 1 College of Computer Science and Technology, Changchun University, Changchun 130022, China; kuangzhejun@ccu.edu.cn (Z.K.); 221501479@mails.ccu.edu.cn (L.G.); 221501471@mails.ccu.edu.cn (J.W.); wangl95@ccu.edu.cn (L.W.); 231501506@mails.ccu.edu.cn (K.G.); 2 Key Laboratory of Intelligent Rehabilitation and Barrier-Free for the Disabled, Ministry of Education, Changchun 130022, China; 3 Jilin Provincial Key Laboratory of Human Health Status Identification & Function Enhancement, Changchun 130022, China

**Keywords:** electroencephalogram, signal analysis and processing, convolutional neural network, seizure electrode sites, seizure onset zones

## Abstract

Background: The localization of seizure onset zones (SOZs) is a critical step before the surgical treatment of epilepsy. Methods and Results: In this paper, we propose an SOZ detection method based on convolutional neural networks and EEG signals. This method aims to locate SOZs through the seizure status of each channel in multi-channel EEG signals. First, we preprocess the data with filtering, segmentation, resampling, and standardization to ensure their quality and consistency. Then, the single-channel UCI epilepsy seizure recognition dataset is used to train and test the convolutional neural network (CNN) model, achieving an accuracy of 98.70%, a sensitivity of 97.53%, and a specificity of 98.98%. Next, the multi-channel clinical EEG dataset collected by a hospital is divided into 21 single-channel site datasets and input into the model for detection, and then the seizure results of 21 sites per second are obtained. Finally, the seizure sites are visualized through the international 10–20 system electrode distribution map, diagrams of the change process of the seizure sites during seizures are drawn, and patients’ SOZs are located. Conclusions: Our proposed method well classifies seizure and non-seizure data and successfully locates SOZs by detecting the seizure results of 21 sites through a single-channel model. This study can effectively assist doctors in locating the SOZs of patients and provide help for the diagnosis and treatment of epilepsy.

## 1. Introduction

Epileptic seizures are recurring seizures caused by abnormal discharges of neurons in the brain. Patients may show different symptoms, including abnormal behaviors, feelings, and even a loss of consciousness. According to statistics from the World Health Organization (WHO), epilepsy affects approximately 50 million people worldwide [1]. Among epilepsy patients, 70% can manage seizures through drug treatment, and 30% are drug-resistant. The best treatment method is to surgically remove the epileptogenic zone [2,3]. Seizure onset zones refer to the area of the cerebral cortex that actually causes seizures, and they are considered surrogate markers for the epileptogenic zone [4]. Therefore, it is crucial to accurately locate SOZs before performing surgery.

Electroencephalogram (EEG) is a tool used to detect seizures and localize SOZs, playing an important role in epilepsy diagnosis. EEG signals are usually continuous and complex. Doctors need to view a large amount of EEG data during epilepsy diagnosis, which is a difficult and very time-consuming process [5,6]. Therefore, research on automatically extracting EEG signal features for epilepsy detection and SOZ localization is necessary.

The localization of seizure areas is based on the International 10–20 Electrode System [7]. Doctors can determine the type of seizure by locating the SOZs. Seizure types can be divided into focal seizures, generalized seizures, and unknown seizures based on their characteristics [8,9]. Generalized seizures have seizure abnormal electrical activity in multiple areas of both hemispheres of the brain. Focal seizures originate in a network area limited to one hemisphere of the brain, spread to surrounding normal areas, and may rapidly spread to both hemispheres of the brain.

In the field of epilepsy, machine learning and deep learning have been widely used to process and analyze EEG data to detect seizures and have achieved remarkable results. Sadam et al. [10] proposed a hybrid convolutional neural network and support vector machine model for seizure detection with EEG signals. Hermawan et al. [11] used three models, a convolutional neural network (CNN), long short-term memory network (LSTM), and hybrid CNN-LSTM, for seizure detection, and the CNN model performed best. Dong et al. [12] proposed an automatic detection method for seizures using a temporal convolutional network combined with a bidirectional long short-term memory network. Shi et al. [13] proposed a double attention-based convolutional neural network for seizure detection, with attention-based CNN model feature extraction to obtain the final classification results. Convolutional neural networks are the most commonly used model for seizure detection. Researchers have made improvements based on convolutional neural networks, and these networks have achieved better classification accuracy but often have higher training costs [14].

Researchers are also committed to researching methods and techniques that utilize machine learning and deep learning to achieve SOZ localization. Elahian et al. [15] studied a method based on the phase-locking value (PLV) in electrocorticography (ECoG), using features extracted from the PLV and logistic regression to build a model for the classification of seizure and non-seizure zones. Siddiqui et al. [16] proposed a method to achieve rapid seizure detection and localization on the ECoG dataset through two decision forest classifiers: SysFor and Forest CERN. Johnson et al. [17] localized SOZs by training a multi-channel one-dimensional convolutional neural network on stereoelectroencephalogram cortical evoked potentials. In addition, high-frequency oscillations (HFOs) are considered biomarkers of SOZs [18,19]. Wu et al. [3] proposed an unsupervised detector that can automatically detect HFOs in intracranial electroencephalogram (iEEG) signals. Wan et al. [20] proposed a method to automatically detect HFOs in iEEG signals based on singular value decomposition and improved fuzzy c-means clustering. Both methods use detected HFO concentrations to locate SOZs. Zhao et al. [21] introduced an EEG augmentation method to solve the problem of iEEG data imbalance and classify SOZ and non-SOZ data through a one-dimensional convolutional neural network model. Yang et al. [22] proposed a method for epileptic focus localization based on multi-modal EEG (iEEG and scalp EEG) and transfer learning, which can achieve the classification of epileptic and non-epileptic channels. Zhao et al. [23] achieved high-performance results for SOZ and non-SOZ data classification by using labeled iEEG data and support vector machine, a fully connected neural network, and a convolutional neural network as classification models.

In past studies, machine learning and deep learning methods have mainly focused on feature analyses of intracranial signals to locate SOZs. In contrast, studies on locating SOZs based on scalp EEG (sEEG) signals are scarce. Tibdewal et al. [24] distinguished epileptic and non-epileptic EEG signals through the variance and values of five different entropy estimators, and they detected and localized the most affected channels and regions in multi-channel seizure signals. Mansouri et al. [25] proposed a method to detect seizures by calculating the distance and Pearson correlation coefficients between neighboring electrodes in sEEG and by using the connection ratio and strength of the correlation network to locate the seizure origin. De et al. [26] used an independent component analysis to decompose the sEEG signal during seizures, and they combined it with the source imaging method to achieve the localization of SOZs. Traditional machine learning algorithms or statistical methods used for analysis and localization cannot adequately learn the complex patterns and underlying features in EEG signals, and the generalization ability of the methods may be limited. Craley et al. [27] proposed an SZTrack architecture for scalp EEG seizure detection and localization, which combines a convolutional neural network encoder and a recurrent neural network. Convolutional neural networks can automatically learn and extract features from input data without the need for manually designed feature extractors, thus being able to capture complex features that traditional methods may overlook.

In this paper, we propose an SOZ detection method based on convolutional neural networks and EEG signals. This method aims to use multi-channel sEEG data to detect multiple scalp seizure sites during seizures and locate the seizure areas based on the location of the seizure sites per second. We preprocess the data, including filtering, segmentation, resampling, and standardization, to ensure their quality and consistency. The single-channel dataset is standardized and used to train the CNN model. After filtering, segmentation, resampling, and standardization, the multi-channel EEG data are divided into 21 single-channel site datasets, which are input into the model to detect the seizure results of the 21 scalp sites. This study realizes the visualization of multiple seizure sites per second. By analyzing the change process of seizures at electrode sites, we can locate SOZs and determine the type of seizure.

The innovations and contributions of this paper can be summarized as follows:

- The multi-channel EEG data of clinical epilepsy patients collected in a hospital are divided into 21 single-channel site datasets based on the channel names. They are input into 21 trained CNN models for detection, and the per-second seizure results of the 21 electrode sites are obtained. This determines at which electrode sites seizures occurred.
- On the international 10–20 system electrode distribution map, the seizure results of the 21 electrode sites per second are visualized. The change process of the patients’ seizure sites, from the beginning of the seizure to its enhancement and gradual weakening near the end, is drawn. This achieves the localization of the patients’ SOZs.
- The localization of SOZs can assist doctors in more accurately diagnosing patients’ seizure types, such as generalized seizure or focal seizure.

## 2. Methods

### 2.1. Framework

**Figure 1:**
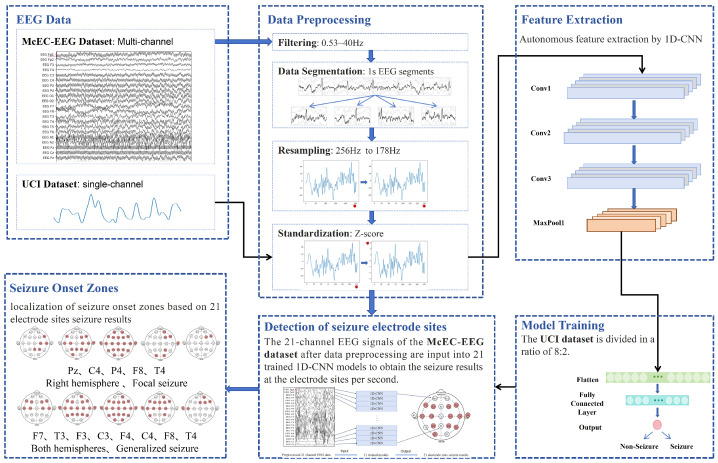
The framework of the SOZ detection method based on convolutional neural networks and EEG signals.

The SOZ detection method proposed in this paper, based on a CNN and EEG signals, is shown in Figure 1. The method mainly includes preprocessing EEG data, autonomously extracting features using a CNN, training and testing the model with single-channel datasets, detecting seizure sites with multi-channel datasets, visualizing the seizure process at sites, and locating SOZs. Data preprocessing includes filtering, segmentation, resampling, and standardization to ensure their quality and consistency. The single-channel UCI dataset is standardized and used to train the CNN model. The CNN model autonomously extracts discriminative features from the EEG signal through the convolutional layer, making up for the shortcomings of manual feature extraction. After filtering, segmentation, resampling, and standardization, the multi-channel McEC-EEG dataset is divided into 21 single-channel site datasets, which are input into the model to detect the seizure results per second at the 21 sites. The scalp seizure site results detected every second are visualized on the international 10–20 system electrode distribution map, and the preliminary localization of SOZs is achieved by analyzing the change process of site seizures.

### 2.2. Experimental Settings

Our experiments were conducted on a computer equipped with a 12th Gen Intel(R) Core(TM) i5-12500H @2.50 GHz processor, 16G RAM, an NVIDIA RTX 3050 graphics card, and a 64-bit Windows 11 operating system.The computer is manufactured by Lenovo, a company located in Beijing, China. The software tools used for the experiments and result analysis were Pycharm 2023.2.4 and Anaconda 2021.05. The experiments were implemented in Python 3.11.2, Conda 4.14.0, and some important libraries were used, such as Pandas 1.5.3, Tensorflow 2.12.0, Keras 2.12.0, Scikit-Learn 1.2.2, Numpy 1.23.5, and Mne 1.3.1.

### 2.3. EEG Database

#### 2.3.1. UCI Epilepsy Seizure Recognition Dataset (UCI Dataset)

The epileptic seizure recognition dataset was obtained from the UCI Machine Learning Library, and it is a preprocessed and reorganized version of the University of Bonn epilepsy seizure dataset. The dataset consists of 5 different sub-datasets, each containing 100 data segments, which were manually cut from a longer multi-channel EEG while removing some possible interference, including muscle and eye movement artifacts. The bandpass filter was set to 0.53–40 Hz [28]. The time length of each data segment is 23.6 s, and the sampling frequency is 173.61 Hz; thus, the corresponding time series is sampled into 4097 data points, and each data point is an EEG recording value at a different time point. Every 4097 data points are divided into 23 blocks, so the dataset has 11,500 rows of data. Each row of data (lasting 1 s) contains 179 attributes, including 178 data points, and the last column label value is y{1, 2, 3, 4, 5}. The 5 sub-datasets are labeled into 5 categories. Label 5 indicates EEGs recorded with eyes open, label 4 indicates EEGs recorded with eyes closed, label 3 indicates EEGs recorded in the healthy brain area after the tumor area is identified, label 2 indicates EEGs recorded in the area where the tumor is located, and label 1 records seizure activity [29]. Each category contains 2300 signals. Among them, the data belonging to categories 2, 3, 4, and 5 are non-seizure data, and only category 1 records epileptic seizure activity. The complete dataset is available at https://www.kaggle.com/datasets/harunshimanto/epileptic-seizure-recognition (accessed on 25 March 2024).

#### 2.3.2. Multi-Channel Epilepsy Clinical Dataset (McEC-EEG Dataset)

The multi-channel epilepsy clinical dataset contains continuous 4-hour scalp EEG data of four clinical patients with epilepsy recorded during the interictal, preictal, and ictal periods, saved as edf files. The sampling frequency is 256 Hz. In this paper, we selected 21 channels of EEG signals (Fp1, Fp2, F3, F4, C3, C4, P3, P4, O1, O2, F7, F8, T3, T4, T5, T6, A1, A2, Fz, Cz, and Pz) for experiments, removing T1 and T2 signals, electrocardiogram (ECG) signals, electromyography (EMG) signals, and other irrelevant signals. The electrode placement during the EEG signal collection process complies with the international 10–20 electrode system. The seizure time, number of seizures, seizure type, and seizure regions in this dataset were annotated by a hospital neurosurgeon. The detailed information of the McEC-EEG dataset is shown in Table 1.

### 2.4. Data Preprocessing

In this study, the UCI dataset includes intracranial and scalp signals. The McEC EEG dataset was obtained from the scalp. However, iEEG and sEEG have similarities. Both iEEG and sEEG record brain waves of different frequencies, such as delta, theta, alpha, beta, and gamma waves. When a seizure occurs, both iEEG and sEEG records show specific waveform features, such as spike waves, sharp waves, spike and slow waves, and sharp and slow waves. We used appropriate data preprocessing methods, such as filtering, resampling, and standardization, to better eliminate the differences between the data of the two datasets. Therefore, we were still able to successfully train an effective model using the UCI dataset.

Our methed detects seizures or non-seizures, which is a binary classification problem, so the UCI dataset was converted into a binary classification dataset. The label value y of the non-seizure data with labels 2, 3, 4, and 5 was set to 0, and the label value y of the seizure data with category 1 remained unchanged at 1.

Since there are significant differences between the UCI dataset and the McEC-EEG dataset in terms of data structure, data format, data distribution, etc., we performed different preprocessing steps on the two datasets to ensure data quality and consistency. The McEC-EEG dataset was filtered, segmented, resampled, and standardized, and the UCI dataset was standardized. In this way, we obtained two datasets with a uniform format and reliable quality.

#### 2.4.1. Filtering

In an EEG signal, the important information for seizure detection is between the 0 and 40 Hz frequency bands, so a band-pass filter of 0.53–40 Hz is applied in the UCI dataset [30]. In this paper, we take the same approach when filtering the EEG signals of all channels in the McEC-EEG dataset. We set a band-pass filter of 0.53–40 Hz to limit the frequency range between 0.53 and 40 Hz, consistent with the UCI data.

#### 2.4.2. Data Segmentation

The 21 channels in the McEC-EEG dataset correspond to the 21 electrode sites of the international 10–20 system electrode distribution map [31,32], which represents the entire scalp area. Therefore, the filtered McEC-EEG data were divided. Firstly, the 21 channels of EEG signals were selected, and the T1 and T2 channels, ECG signals, EMG signals, and other irrelevant signals were removed. Secondly, the multi-channel EEG signal was divided into 21 single-channel site datasets, and the channel names correspond to the 21 electrode sites. Finally, the EEG signals of each channel dataset were divided into non-overlapping signal segments with a time of 1 s as samples [33].

#### 2.4.3. Resampling

Resampling is a common signal processing technique used to adjust the sampling rate of a signal to suit specific application needs or to synchronize with other signals. After data segmentation, the McEC-EEG signal was divided into samples with a length of 1 s and containing 256 sampling points. The samples in the UCI dataset have 178 sampling points per second. Different sampling rates lead to inconsistencies between datasets, affecting subsequent data analysis. In order to ensure the consistency and comparability of the data and eliminate the bias that may be caused by different sampling rates, we resampled the McEC-EEG dataset to make its sampling rate consistent with the UCI dataset [34,35,36]. Therefore, the McEC-EEG dataset was resampled to 178 Hz.

#### 2.4.4. Standardization

In order to ensure the same scale between the different datasets and avoid biasing the results based on the amplitude of the signal [37], we used the Z-score standardization method to process the UCI and McEC-EEG data samples. The data samples were converted to a state where the mean was 0 and the variance was 1. The Z-score standardization formula is as follows:

(1) $$x^{\prime }=\frac{x-\mu }{\sigma }$$

Here, *x* is a certain feature value of the original data sample; μ is the mean value of the feature column; σ is the standard deviation of the feature column; and $x^{\prime }$ is the standardized data.

### 2.5. CNN Autonomous Feature Extraction

**Figure 2:**
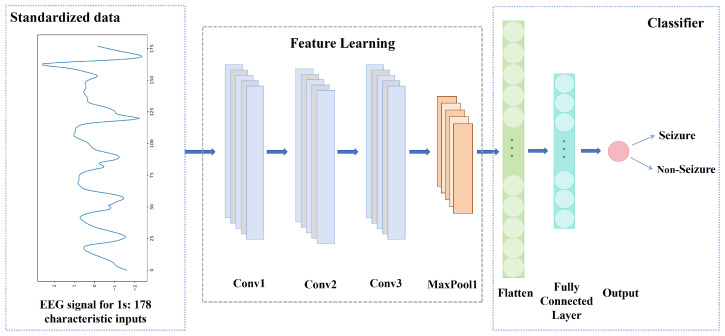
1D-CNN: the model of feature extraction and classification.

In this paper, we use a one-dimensional convolutional neural network (1D-CNN) to autonomously extract features from the EEG signal data [38,39], as shown in Figure 2. The parameters of the model are shown in Table 2. The inputs of the model are EEG samples with a length of 1 s and a number of 178 feature values. The model uses three convolutional layers to perform autonomous feature extraction on EEG signals. Each of the three convolutional layers uses 20 convolution kernels of size 5 × 1 to convolve the input data with a step size of 1, and the output results are passed to the next layer in sequence. The output of each convolutional layer uses the Relu activation function. After each convolutional layer, a dropout layer with a parameter of 0.5 is added, randomly discarding the output of 50% of the neurons. The pooling window size of the maximum pooling layer is 2. By selecting the maximum feature value, the dimension of the convolutional layer output is reduced to prevent model overfitting. A flatten layer is used to flatten the data into a one-dimensional vector and feed it into the first fully connected layer. The first fully connected layer has 50 neurons and uses the ReLU activation function for further extraction and combination of features. The second fully connected layer is used for output and has one neuron. The sigmoid activation function is used to output the probability that the sample is a seizure or non-seizure sample, and the threshold is set to 0.5.

The following is a detailed introduction to the CNN layers and activation functions used in this article.

Convolutional layers are the core component of the CNN. The convolution layer performs a convolution operation on the input data of the previous layer, learns different features of the data through the convolution kernel, and passes the extracted feature information to the next layer [40]. This layer uses multiple convolution kernels to perform one-dimensional convolution on the input data to obtain more feature maps [41]. The calculation principle of the k-th feature map in the convolutional layer is as follows:

(2) $$Y_{k}=f\left(W_{k}*X+b_{k}\right)$$

Here, *X* is the input of the convolutional layer, and $Y_{k}$ is the output of the *k*-th convolution kernel. $W_{k}$ and $b_{k}$ represent the weight and bias of the convolution kernel, respectively, and ∗ is the convolution computation. The function $f\left(\cdot \right)$ is a nonlinear activation function, and the convolutional layer in this experiment uses the Relu activation function.

The max pooling layer processes the input feature map through down-sampling, which can effectively reduce the dimension of the output feature map and reduce the calculation amount and parameter amount of the model [41]. The max pooling layer slides a fixed-size pooling window on the input feature map and selects the maximum value within the window as the output [42]. The principle is shown in Equation (3). The maximum pooling window for this experiment is 2 × 1.

(3) $$Y_{i}=max\left(X_{i}\right)$$

Here, $X_{i}$ represents the input feature value of the *i*-th pooling window, and $Y_{i}$ represents the *i*-th output feature value. The function $max\left(\cdot \right)$ takes the maximum value of all features in the window.

The dropout layer randomly discards some neurons with a certain probability during the training process to prevent overdependence between neurons and effectively reduce the risk of overfitting. In this experiment, the parameter of the dropout layer is set to 0.5.

The flatten layer is used to flatten the input multi-dimensional feature map into a one-dimensional vector so that it can be passed to the fully connected layer for further processing.

The fully connected layer connects all neurons in the previous layer to each neuron in the current layer [43]. It can effectively integrate all features of the previous layer to form a global feature representation to provide support for the final classification. The calculation principle of the fully connected layer is as follows:

(4) $$Y=f\left(WX+b\right)$$

Here, *Y* is the output of the fully connected layer, and *X* is the input. *W* and *b* represent the weight and bias of the fully connected layer, respectively. The function $f\left(\cdot \right)$ is a nonlinear activation function.

The output layer node corresponds to the output variables [44]. It receives the global feature representation from the previous layer, applies an activation function to transform the features into the model’s final result, and outputs it.

The Relu activation function is a nonlinear activation function used to implement nonlinear operations in neural networks. The characteristic of the Relu function is that it outputs the *x* value when input *x* is a positive number, and the output is zero when *x* is a negative number. The function is defined as follows:

(5) $$f\left(x\right)=max\left(0,x\right)$$

Here, the function $max\left(0,x\right)$ takes the maximum value between 0 and input *x*.

The sigmoid activation function maps the input to an output value ranging from 0 to 1. It is often used as the activation function of the output layer in binary classification to generate a probability value between 0 and 1, indicating the probability that the sample belongs to a certain category. The function is defined as follows:

(6) $$\sigma \left(x\right)=\frac{1}{1+e^{-x}}$$

Here, *x* is the input, σ(x) is the output, and *e* is the base value of the natural logarithm.

### 2.6. Model Evaluation

In this paper, we use three statistical indicators, Accuracy, Sensitivity, and Specificity, to evaluate the performance of the model in detecting seizures. The calculation formula is defined as follows:

(7) $$Accuracy=\frac{\mathrm{TP}+\mathrm{TN}}{\mathrm{TP}+\mathrm{FN}+\mathrm{TN}+\mathrm{FP}}\times 100\%$$

(8) $$Sensitivity=\frac{\mathrm{TP}}{\mathrm{TP}+\mathrm{FN}}\times 100\%$$

(9) $$Specificity=\frac{\mathrm{TN}}{\mathrm{TN}+\mathrm{FP}}\times 100\%$$

Here, TP represents the number of samples for which both the model detection result and the label are seizures. TN indicates the number of samples whose model detection results and labels are non-seizures. FP indicates the number of samples that the model detects as seizures but are actually non-seizures. FN indicates the number of samples that the model detects as non-seizures but are actually seizures. Accuracy indicates the ratio of correctly classified samples to total samples. Sensitivity represents the ratio of correctly predicted seizure samples to actual seizure samples. Specificity represents the ratio of correctly predicted non-seizure samples to actual non-seizure samples.

### 2.7. Model Training

We divided the standardized UCI dataset into a training set, validation set, and test set and used the 1D-CNN model to automatically extract and learn data features. When the training, validation, and test sets are randomly divided, the result of the validation phase depends on the data used during the validation and the data used in the training phase. The validation result will be unreliable [45]. Therefore, we used 10-fold cross-validation. In the experiment, the training and validation sets contained 80% of the data, and the test set contained 20% of the data. According to the 10-fold cross-validation method, the training and validation subsets were randomly divided. The test set was used to further evaluate the performance of the CNN model.

The synthetic minority oversampling technique (SMOTE) is a method used to solve the problem of class imbalance in datasets. The SMOTE algorithm generates new synthetic samples by interpolating between minority class samples, thereby increasing the number of minority class samples. We used the SMOTE algorithm on the training and validation sets to solve the problem of class imbalance in the UCI data.

Our method detects seizures and non-seizures, which is a binary classification problem. Therefore, we chose the binary cross-entropy loss function and the adaptive moment estimation optimizer and set the training epoch to 200. To avoid overfitting, we introduced an early stopping mechanism and set the patience to 16. We conducted multiple experiments to test the impact of different parameters on model performance. We averaged the results of the 10-fold cross-validation, and the results are shown in Table 3. By comparing these results, we selected the best experimental parameters: a learning rate of 0.001, a batch size of 256, and a pool size of 2.

Under the above parameters, the average accuracy of the model in the 10-fold cross-validation is 99.13%, the average sensitivity is 99.32%, and the average specificity is 98.94%. We use sensitivity as the main evaluation metric to ensure that the model can effectively detect seizures [46,47]. The results of the best performing model are as follows: the accuracy is 99.12%, the sensitivity is 99.72%, and the specificity is 98.53%. We select this model for testing on the test set.

### 2.8. Detecting Seizure Sites Using 1D-CNN Models

During a seizure, abnormal electrical activity occurs first in the epileptogenic zone. After passing through neurons in the brain, electrical signals are transmitted to the scalp. At this time, several channels or all channels in the scalp EEG become chaotic high-frequency signals. After the end of the seizure, the signals become flat [48]. Therefore, in this paper, we determine SOZs by analyzing the seizure status of each channel.

The McEC-EEG dataset contains the EEG signals of four clinical patients. We select continuous EEG signal segments containing one seizure for each patient as site detection data. After data preprocessing, 21 site datasets named after the channel are obtained, in which each sample is 1 s of the EEG signal.

For the detection of seizure sites, 21 site datasets are input into 21 identical 1D-CNN models trained on the UCI dataset, thereby obtaining the seizure results of 21 sites per second, as shown in Figure 1. The results correspond to the electrode sites of the international 10–20 electrode system. Then, we can determine at which electrode sites the seizure occurred and initially determine SOZs and seizure types.

## 3. Results

### 3.1. UCI Data Seizure Classification Results

Our method is based on the UCI dataset, which was used to train, validate, and test the model. The model achieved an accuracy of 98.70%, a sensitivity of 97.53%, and a specificity of 98.98% on the test set, indicating that it has a good generalization ability and can well classify new samples.

We conducted comparative experiments using machine learning methods with manual feature extraction. We used discrete wavelet transform (DWT) combined with random forest and Extra Tree [49] for seizure detection on the UCI dataset. The results are shown in Table 4. In comparison, our 1D-CNN model performs better.

### 3.2. McEC-EEG Data Seizure Sites and SOZ Detection Results

**Figure 3:**
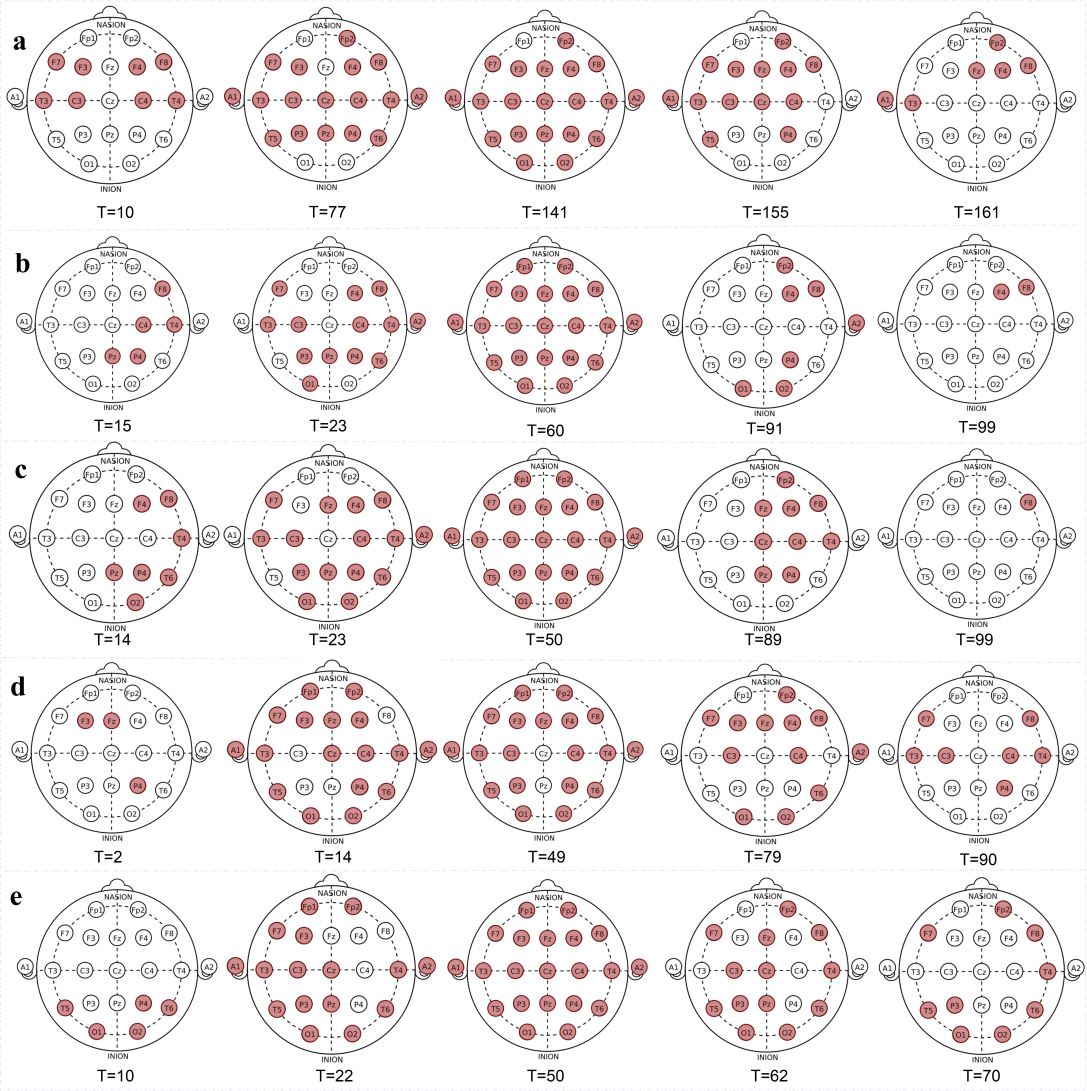
The changing process of site discharge during seizures in four patients. The red sites represent the predicted seizure sites, and T represents the seconds of the seizure period. Figure (**a**) shows the changing process of site discharge during the seizure of patient 1. Figure (**b**) shows the changing process of site discharge during the first seizure of patient 2. Figure (**c**) shows the second seizure of patient 2. Figure (**d**) shows the changing process of site discharge during the seizure of patient 3. Figure (**e**) shows the changing process of site discharge during the seizure of patient 4.

During seizures, the abnormal electrical activity at scalp sites changes continuously with time. Through 21 1D-CNN model detection, we obtained per-second seizure results for 21 sites in the patients’ continuous EEG signals. We achieved visualization of the seizure change process at each site from the beginning of the seizure to near the end of the seizure, thereby locating the patients’ SOZs. We constructed a visualization diagram of the seizure results at five interval time nodes to represent the entire change process of the seizures, as shown in Figure 3. The detection results of the SOZs are shown in Table 5.

Patient 2 had two seizure signals. We show the change process of patient 2’s seizure sites from the beginning of the seizure to near the end of the seizure in Figure 3b. When patient 2’s first seizure started, it occurred in Pz, C4, P4, F8, and T4 at the 15th second. These sites are located on the right side of the scalp and quickly spread to the surrounding area, gradually spreading to the whole scalp. Near the end of the seizure, there were still seizures at sites F4 and F8 on the right side of the brain. Therefore, the SOZ of patient 2 is located in the right hemisphere of the brain.

In order to verify the SOZ of patient 2, we selected the second seizure data with a longer interval to conduct the experiment again, as shown in Figure 3c. The seizure of patient 2 spread from the right side of the brain to the entire scalp. Near the end of the seizure, the right side of the brain still showed seizure abnormal electrical activity at F8. Although the site detection results of the two seizures are different, the seizure sites in the initial seizure and near the end of the seizure are distributed in the right hemisphere of the brain. Therefore, it is more certain that the SOZ of patient 2 is in the right hemisphere of the brain.

Figure 3 shows the change process of seizure sites during the seizure period of patients 1, 3, and 4. Patient 1 had seizures in the F7-F3-C3-T3 area of the left hemisphere and the F4-C4-T4-F8 area of the right hemisphere at the 10th second. Near the end, seizures were still detected in the A1-T3 area and the Fp2-Fz-F4-F8 area. When patient 3 had a seizure, it first started at the F3, Fz, and P4 sites and gradually spread. When the seizure was nearing its end, seizure sites were detected in the F7-T3-C3 areas of the left hemisphere and the C4-P4-T4-F8 areas of the right hemisphere. During patient 4’s seizure, seizure sites were detected in the occipital (O1, O2), posterior temporal (T5, T6), and right hemisphere parietal (P4) of the brain at the 10th second. As the seizure progressed, the seizure phenomenon gradually spread throughout the whole scalp. When the seizure subsided near the end, there were still seizure sites on the scalp of both hemispheres. We found that the three patients had seizure abnormal electrical activity in multiple areas of both hemispheres of the brain, so the SOZs of the three patients are both hemispheres of the brain.

Our SOZ detection results are consistent with the annotated seizure regions. Patients 1, 3, and 4 had seizures in multiple regions of the brain, and the initial seizure region of patient 2 was in the right hemisphere of the brain. Our method can perform the localization of SOZs.

### 3.3. McEC-EEG Data Seizure Type Results

Seizure types are mainly divided into focal seizures and generalized seizures. The seizure process of the four patients in the McEC-EEG dataset can assist doctors in diagnosing the seizure type of each patient.

The origin of seizure abnormal electrical activity in focal seizures is primarily confined to areas in one hemisphere of the brain. According to Figure 3, the two seizures of patient 2 both started in the right hemisphere of the brain, and then the discharge activity spread to other areas. Based on this discharge pattern, it can be inferred that the seizure type of patient 2 is a focal seizure.

In generalized seizures, seizure abnormal electrical activity appears in multiple areas of the brain at the same time or nearly simultaneously and is widely distributed. According to Figure 3, patients 1, 3, and 4 had seizures in multiple areas of both hemispheres of the brain during their initial stages. Therefore, it can be inferred that the seizure types of the three patients are generalized seizures.

## 4. Discussion

Our method is compared with epilepsy detection research methods based on UCI datasets proposed in recent years. The results are shown in Table 6. The selected epilepsy detection methods all deal with the binary classification problem of seizures and non-seizures to ensure the fairness and accuracy of the comparison results. The results show that, in terms of overall performance, our proposed 1D-CNN model outperforms most of the compared methods. Moreover, compared with other deep learning methods, its network structure is simple. We use sensitivity as the main evaluation indicator. The proposed method has a high reliability in the binary classification problem of identifying seizures and non-seizures, which can meet our requirements for the further detection of SOZs.

**Figure 4:**
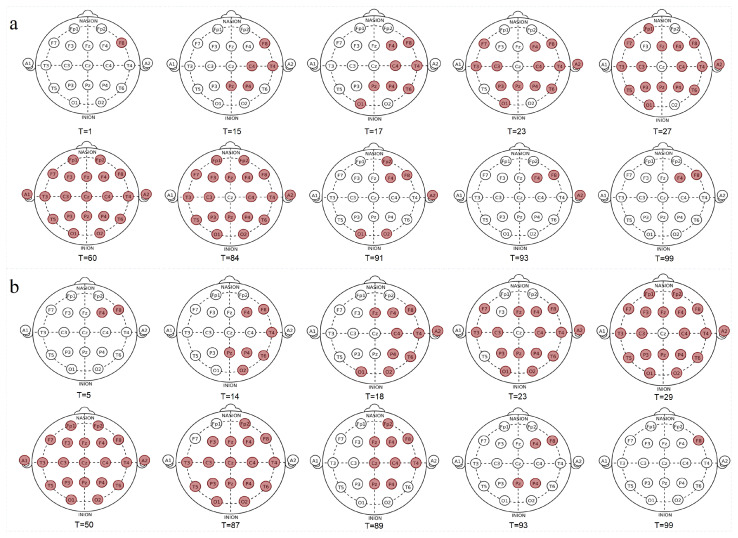
The change process of the seizure sites in patient 2, from the beginning of the seizure, to the intensification of the seizure and spread to the whole scalp, to the gradual weakening of the seizure, and to the near end of the seizure. The red sites represent the sites predicted to be seizure sites, and T represents the seconds of the seizure period. Panel (**a**) shows the first seizure, and panel (**b**) shows the second seizure.

The method proposed in this paper determines the location of SOZs by detecting seizure sites. We conducted an in-depth analysis of the two seizure site detection results for patient 2. Figure 4 shows in detail the change process of the seizure sites in patient 2, from the beginning of the seizure to the intensification of the seizure and spread to the whole scalp, to the gradual weakening of the seizure, and to near the end of the seizure. It clearly shows that patient 2’s two seizures originated in the right hemisphere of the brain and rapidly spread to the left hemisphere. As the seizure intensified, an obvious seizure phenomenon appeared throughout the scalp area. Then, the seizure abnormal electrical activity gradually decreased and eventually disappeared in the right hemisphere of the brain.

The epileptogenic zone reflects the site of the beginning of the seizures and of their primary organization [60]. The first seizure of patient 2 originated from the F8 site and eventually disappeared at the F4 and F8 sites of the right hemisphere. The second seizure originated from the F4 and F8 sites and disappeared at the F8 site of the right hemisphere. In the right hemisphere, F4 and F8 are located in the frontal lobe [61]. Therefore, it can be further inferred that the patient’s epileptogenic zone is in the frontal lobe of the right hemisphere of the brain.

In this paper, we used sEEG signals to locate the seizure region. sEEG signals are recorded by placing multiple surface electrodes on the patient’s scalp. It is relatively simple and safe. However, the spatial resolution of sEEG is low and cannot provide accurate information related to specific structures in the brain. Since iEEG signal acquisition requires electrodes to be implanted into the patient’s brain, this process is costly and accompanied by risks such as bleeding, infection, and death [62]. Therefore, the use of invasive electrodes is justified only when noninvasive methods deliver evidence that the epileptogenic zone is potentially resectable [63]. The method that we proposed can provide doctors with a region to consider for SOZs. Doctors can combine the patient’s clinical manifestations and functional magnetic resonance imaging and even place fewer intracranial electrodes to further narrow the scope of the SOZ and improve the accuracy and effectiveness of surgical treatment.

This study followed ethical guidelines to ensure strict protection of data privacy and security during the research process. We ensured that the EEG data were used only for this study.

## 5. Conclusions and Future Work

In this paper, we propose a detection method for seizure onset zones based on convolutional neural networks and EEG signals. The UCI epilepsy seizure recognition dataset was used to train and test the 1D-CNN model, achieving an accuracy of 98.70%, a sensitivity of 97.53%, and a specificity of 98.98%. The multi-channel McEC-EEG dataset was divided into 21 single-channel site datasets according to channel names and then input into 21 trained 1D-CNN models for detection, so the seizure results of 21 electrode sites per second were obtained. By combining the seizure results from multiple electrode sites, we preliminarily located patients’ SOZs, thereby providing strong support for the diagnosis of epilepsy. The international 10–20 electrode system was used to visualize the seizure electrode sites. We drew diagrams of the change process of seizure sites during the patients’ seizures, and they clearly show the process of the seizure starting, intensifying, and gradually weakening near the end, thus providing doctors with more intuitive and dynamic seizure information.

In future work, we plan to utilize existing seizure onset points and scalp SOZs, combined with magnetic resonance imaging (MRI) technology, to calculate the Euclidean distance between multiple seizure onset points and various intracranial tissue structures and further achieve an in-depth localization of intracranial epileptogenic zones. We will develop an automatic SOZ detection system incorporating the proposed method to assist doctors in more accurately locating SOZs to improve the effectiveness of patient treatment.

## Figures and Tables

**Table 1:** Detailed information of the McEC-EEG dataset.

Patient	Seizure Type	Initial Seizure Region	Number of Seizures	Seizure EEG Length (s)
1	Generalized seizure	Multiple regions in both hemispheres	1	165
2	Focal seizure	Right hemisphere	2	100 100
3	Generalized seizure	Multiple regions in both hemispheres	1	95
4	Generalized seizure	Multiple regions in both hemispheres	1	80
Total	-	-	5	540

**Table 2:** Model-related parameters.

Layer	Kernel Channel Size	Kernel Size	Activation Function	Output Shape
Conv1	20	5 × 1	Relu	174 × 20
Conv2	20	5 × 1	Relu	170 × 20
Conv3	20	5 × 1	Relu	166 × 20
Maxpool1	-	2 × 1	-	83 × 20
Flatten layer	-	-	-	1660
Fully connected layer	-	-	Relu	50
Output layer	-	-	Sigmoid	1

**Table 3:** Model performance with different parameters.

Learning Rate	Batch Size	Pool Size	val_Accuracy (%)	val_Sensitivity (%)	val_Specificity (%)
0.01	128	2	96.99	94.65	99.31
		5	93.29	86.97	99.52
	256	2	97.14	94.70	99.52
		5	95.52	91.64	99.43
0.001	128	2	99.09	99.24	98.94
		5	98.94	98.86	99.02
	256	2	99.13	99.32	98.94
		5	98.80	98.68	98.90
0.0001	128	2	98.75	98.83	98.67
		5	98.27	97.87	98.66
	256	2	98.47	98.26	98.69
		5	97.94	97.25	98.63

**Table 4:** Seizure classification results on UCI dataset.

Method	Accuracy (%)	Sensitivity (%)	Specificity (%)
DWT + Random Forest	96.78	87.50	96.66
DWT + Extra Tree	97.70	93.00	98.27
1D-CNN	98.70	97.53	98.98

**Table 5:** Detection results of the SOZs.

Patient	Seizure Onset Zones
1	Both hemispheres
2 (1)	Right hemisphere
2 (2)	Right hemisphere
3	Both hemispheres
4	Both hemispheres

**Table 6:** Comparison results of seizure detection methods based on the UCI dataset.

Authors	Methods	No. of Layers	Accuracy (%)	Sensitivity (%)	Specificity (%)
Kunekar et al. [50]	Random Forest	-	97.70	92.80	99.00
Kode et al. [51]	1D CNN	11	99.00	99.00	-
Kunekar et al. [52]	LSTM	3	97.10	-	-
Kr et al. [53]	Multi-dimensional CNN BiLSTM	13	99.61	97.42	99.35
Prakash et al. [54]	Modified Gated Recurrent Unit	5	98.84	97.10	-
Ahmad et al. [55]	1D CNN-BiLSTM + TBPTT	11	99.41	98.99	98.80
Raibag et al. [56]	Naive Bayes	-	96.00	94.00	96.00
Woodbright et al. [57]	CNN	8	98.65	96.29	99.25
Rahman et al. [58]	SVM	-	97.86	93.40	98.98
Shankar et al. [59]	PCA with ANN	-	97.55	91.48	-
Us	1D-CNN	7	98.70	97.53	98.98

## Data Availability

Our experimental code can be found at https://github.com/kuirh/Seizure-Onset-Zones-Detection-Based-on-Convolutional-Neural-Networks-and-EEG-Signals. The McEC-EEG data are not publicly available due to privacy restrictions.

## References

[1] World Health Organization (2019). Epilepsy: A Public Health Imperative.

[2] Elger C.E., Hoppe C. (2018). Diagnostic challenges in epilepsy: Seizure under-reporting and seizure detection. Lancet Neurol..

[3] Wu M., Wan T., Ding M., Wan X., Du Y., She J. (2018). A new unsupervised detector of high-frequency oscillations in accurate localization of epileptic seizure onset zones. IEEE Trans. Neural Syst. Rehabil. Eng..

[4] Rosenow F., Lüders H. (2001). Presurgical evaluation of epilepsy. Brain.

[5] Peng H., Li C., Chao J., Wang T., Zhao C., Huo X., Hu B. (2021). A novel automatic classification detection for epileptic seizure based on dictionary learning and sparse representation. Neurocomputing.

[6] Gao X., Yan X., Gao P., Gao X., Zhang S. (2020). Automatic detection of epileptic seizure based on approximate entropy, recurrence quantification analysis and convolutional neural networks. Artif. Intell. Med..

[7] Homan R.W., Herman J., Purdy P. (1987). Cerebral location of international 10–20 system electrode placement. Electroencephalogr. Clin. Neurophysiol..

[8] Thijs R.D., Surges R., O’Brien T.J., Sander J.W. (2019). Epilepsy in adults. Lancet.

[9] Fisher R.S., Cross J.H., French J.A., Higurashi N., Hirsch E., Jansen F.E., Lagae L., Moshé S.L., Peltola J., Roulet Perez E. (2017). Operational classification of seizure types by the International League Against Epilepsy: Position Paper of the ILAE Commission for Classification and Terminology. Epilepsia.

[10] Sadam S.S.P., Nalini N. (2024). Epileptic seizure detection using scalogram-based hybrid CNN model on EEG signals. Signal Image Video Process..

[11] Hermawan A.T., Zaeni I.A.E., Wibawa A.P., Gunawan G., Hendrawan W.H., Kristian Y. (2024). A multi representation deep learning approach for epileptic seizure detection. J. Robot. Control. (Jrc).

[12] Dong X., Wen Y., Ji D., Yuan S., Liu Z., Shang W., Zhou W. (2024). Epileptic Seizure Detection with an End-to-End Temporal Convolutional Network and Bidirectional Long Short-Term Memory Model. Int. J. Neural Syst..

[13] Shi L., Wang Z., Ma Y., Chen J., Xu J., Qi J. (2024). Double Attention-Based Deep Convolutional Neural Network for Seizure Detection Using EEG Signals. Advanced Intelligent Computing in Bioinformatics.

[14] Liu S., Zhou Y., Yang X., Wang X., Yin J. (2024). A Robust Automatic Epilepsy Seizure Detection Algorithm Based on Interpretable Features and Machine Learning. Electronics.

[15] Elahian B., Yeasin M., Mudigoudar B., Wheless J.W., Babajani-Feremi A. (2017). Identifying seizure onset zone from electrocorticographic recordings: A machine learning approach based on phase locking value. Seizure.

[16] Siddiqui M.K., Islam M.Z., Kabir M.A. (2019). A novel quick seizure detection and localization through brain data mining on ECoG dataset. Neural Comput. Appl..

[17] Johnson G.W., Cai L.Y., Doss D.J., Jiang J.W., Negi A.S., Narasimhan S., Paulo D.L., González H.F., Roberson S.W., Bick S.K. (2022). Localizing seizure onset zones in surgical epilepsy with neurostimulation deep learning. J. Neurosurg..

[18] Jacobs J., Staba R., Asano E., Otsubo H., Wu J., Zijlmans M., Mohamed I., Kahane P., Dubeau F., Navarro V. (2012). High-frequency oscillations (HFOs) in clinical epilepsy. Prog. Neurobiol..

[19] Charupanit K., Sen-Gupta I., Lin J.J., Lopour B.A. (2020). Amplitude of high frequency oscillations as a biomarker of the seizure onset zone. Clin. Neurophysiol..

[20] Wan X., Fang Z., Wu M., Du Y. (2020). Automatic detection of HFOs based on singular value decomposition and improved fuzzy c-means clustering for localization of seizure onset zones. Neurocomputing.

[21] Zhao X., Sole-Casals J., Sugano H., Tanaka T. (2022). Seizure onset zone classification based on imbalanced iEEG with data augmentation. J. Neural Eng..

[22] Yang Y., Li F., Luo J., Qin X., Huang D. (2023). Epileptic focus localization using transfer learning on multi-modal EEG. Front. Comput. Neurosci..

[23] Zhao X., Zhao Q., Tanaka T., Solé-Casals J., Zhou G., Mitsuhashi T., Sugano H., Yoshida N., Cao J. (2023). Classification of the epileptic seizure onset zone based on partial annotation. Cogn. Neurodynamics.

[24] Tibdewal M.N., Dey H.R., Mahadevappa M., Ray A., Malokar M. (2017). Multiple entropies performance measure for detection and localization of multi-channel epileptic EEG. Biomed. Signal Process. Control.

[25] Mansouri A., Singh S.P., Sayood K. (2019). Online EEG seizure detection and localization. Algorithms.

[26] de Borman A., Vespa S., El Tahry R., Absil P.A. (2022). Estimation of seizure onset zone from ictal scalp EEG using independent component analysis in extratemporal lobe epilepsy. J. Neural Eng..

[27] Craley J., Jouny C., Johnson E., Hsu D., Ahmed R., Venkataraman A. (2022). Automated seizure activity tracking and onset zone localization from scalp EEG using deep neural networks. Plos ONE.

[28] Andrzejak R.G., Lehnertz K., Mormann F., Rieke C., David P., Elger C.E. (2001). Indications of nonlinear deterministic and finite-dimensional structures in time series of brain electrical activity: Dependence on recording region and brain state. Phys. Rev. E.

[29] Bhadra R., Singh P.K., Mahmud M. (2024). HyEpiSeiD: A hybrid convolutional neural network and gated recurrent unit model for epileptic seizure detection from electroencephalogram signals. Brain Inform..

[30] Subasi A., Kevric J., Abdullah Canbaz M. (2019). Epileptic seizure detection using hybrid machine learning methods. Neural Comput. Appl..

[31] Jurcak V., Tsuzuki D., Dan I. (2007). 10/20, 10/10, and 10/5 systems revisited: Their validity as relative head-surface-based positioning systems. Neuroimage.

[32] Khazi M., Kumar A., Vidya M. (2012). Analysis of EEG using 10: 20 electrode system. Int. J. Innov. Res. Sci. Eng. Technol..

[33] Vishnu K., Singh N., Hazarika D., Gupta C.N. (2023). Low to High Dimensional Projection of Seizure Electroencephalography Using Recurrent Neural Network. Proceedings of the 2023 IEEE 20th India Council International Conference (INDICON).

[34] Tawhid M.N.A., Siuly S., Li T. (2022). A convolutional long short-term memory-based neural network for epilepsy detection from EEG. IEEE Trans. Instrum. Meas..

[35] Abou-Abbas L., Jemal I., Henni K., Ouakrim Y., Mitiche A., Mezghani N. (2022). EEG oscillatory power and complexity for epileptic seizure detection. Appl. Sci..

[36] Wang D., Ren D., Li K., Feng Y., Ma D., Yan X., Wang G. (2018). Epileptic seizure detection in long-term EEG recordings by using wavelet-based directed transfer function. IEEE Trans. Biomed. Eng..

[37] Van Mierlo P., Carrette E., Hallez H., Raedt R., Meurs A., Vandenberghe S., Van Roost D., Boon P., Staelens S., Vonck K. (2013). Ictal-onset localization through connectivity analysis of intracranial EEG signals in patients with refractory epilepsy. Epilepsia.

[38] Wei Z., Zou J., Zhang J., Xu J. (2019). Automatic epileptic EEG detection using convolutional neural network with improvements in time-domain. Biomed. Signal Process. Control.

[39] Wang X., Wang X., Liu W., Chang Z., Kärkkäinen T., Cong F. (2021). One dimensional convolutional neural networks for seizure onset detection using long-term scalp and intracranial EEG. Neurocomputing.

[40] Zhou M., Tian C., Cao R., Wang B., Niu Y., Hu T., Guo H., Xiang J. (2018). Epileptic seizure detection based on EEG signals and CNN. Front. Neuroinformatics.

[41] Wen T., Zhang Z. (2018). Deep convolution neural network and autoencoders-based unsupervised feature learning of EEG signals. IEEE Access.

[42] Hu W., Cao J., Lai X., Liu J. (2023). Mean amplitude spectrum based epileptic state classification for seizure prediction using convolutional neural networks. J. Ambient. Intell. Humaniz. Comput..

[43] Abiyev R., Arslan M., Bush Idoko J., Sekeroglu B., Ilhan A. (2020). Identification of epileptic EEG signals using convolutional neural networks. Appl. Sci..

[44] Ge J., Zhang S. (2020). Adaptive inventory control based on fuzzy neural network under uncertain environment. Complexity.

[45] Gramacki A., Gramacki J. (2022). A deep learning framework for epileptic seizure detection based on neonatal EEG signals. Sci. Rep..

[46] Emami A., Kunii N., Matsuo T., Shinozaki T., Kawai K., Takahashi H. (2019). Seizure detection by convolutional neural network-based analysis of scalp electroencephalography plot images. Neuroimage Clin..

[47] Avcu M.T., Zhang Z., Chan D.W.S. (2019). Seizure detection using least EEG channels by deep convolutional neural network. Proceedings of the ICASSP 2019-2019 IEEE International Conference on Acoustics, Speech and Sgnal Processing (ICASSP).

[48] Liang W., Pei H., Cai Q., Wang Y. (2020). Scalp EEG epileptogenic zone recognition and localization based on long-term recurrent convolutional network. Neurocomputing.

[49] Irfan M., Siddiqa H.A., Nahli A., Chen C., Xu Y., Wang L., Nawaz A., Subasi A., Westerlund T., Chen W. (2023). An Ensemble Voting Approach with Innovative Multi-Domain Feature Fusion for Neonatal Sleep Stratification. IEEE Access.

[50] Kunekar P., Kumawat C., Lande V., Lokhande S., Mandhana R., Kshirsagar M. (2024). Comparison of Different Machine Learning Algorithms to Classify Epilepsy Seizure from EEG Signals. Eng. Proc..

[51] Kode H., Elleithy K., Almazedah L. (2024). Epileptic Seizure detection in EEG signals using Machine Learning and Deep Learning Techniques. IEEE Access.

[52] Kunekar P., Gupta M.K., Gaur P. (2024). Detection of epileptic seizure in EEG signals using machine learning and deep learning techniques. J. Eng. Appl. Sci..

[53] KR A.B., Srinivasan S., Mathivanan S.K., Venkatesan M., Malar B.A., Mallik S., Qin H. (2023). A multi-dimensional hybrid CNN-BiLSTM framework for epileptic seizure detection using electroencephalogram signal scrutiny. Syst. Soft Comput..

[54] Prakash V., Kumar D. (2023). A modified gated recurrent unit approach for epileptic electroencephalography classification. J. Inf. Commun. Technol..

[55] Ahmad I., Wang X., Javeed D., Kumar P., Samuel O.W., Chen S. (2023). A hybrid deep learning approach for epileptic seizure detection in EEG signals. IEEE J. Biomed. Health Inform..

[56] Raibag M.A., Franklin J.V., Sarkar R. (2022). An Investigation on Epileptic Seizure Classification Using Machine Learning and Multiple Feature Selection Strategies. Proceedings of the 2022 3rd International Conference for Emerging Technology (INCET).

[57] Woodbright M., Verma B., Haidar A. (2021). Autonomous deep feature extraction based method for epileptic EEG brain seizure classification. Neurocomputing.

[58] Rahman A.A., Faisal F., Nishat M.M., Siraji M.I., Khalid L.I., Khan M.R.H., Reza M.T. (2021). Detection of epileptic seizure from EEG signal data by employing machine learning algorithms with hyperparameter optimization. Proceedings of the 2021 4th International Conference on Bio-Engineering for Smart Technologies (BioSMART).

[59] Shankar R.S., Raminaidu C., Raju V.S., Rajanikanth J. (2021). Detection of Epilepsy based on EEG Signals using PCA with ANN Model. J. Phys. Conf. Ser..

[60] Jehi L. (2018). The epileptogenic zone: Concept and definition. Epilepsy Curr..

[61] Homan R.W. (1988). The 10–20 electrode system and cerebral location. Am. J. Eeg Technol..

[62] Burneo J.G., Steven D.A., McLachlan R.S., Parrent A.G. (2006). Morbidity associated with the use of intracranial electrodes for epilepsy surgery. Can. J. Neurol. Sci..

[63] Noachtar S., Rémi J. (2009). The role of EEG in epilepsy: A critical review. Epilepsy Behav..

